# Case of Cephaloma, Cured in the Foot, Subsequently Developed in Various Internal Organs

**Published:** 1846-04

**Authors:** Richard Francis George

**Affiliations:** Senior Surgeon to the Bath Hospital


*Case of Cephaloma, cured in the Foot, subsequently developed in various Internal Organs.* By RICHARD FRANCIS GEORGE, Esq., Senior Surgeon to the Bath Hospital.—A healthy woman, aged 35, in October, 1843, discovered in the hollow of her left foot, a painful purple tumour of the size of a pea, which continued to increase for six weeks, when, the skin cracking over it, she first sought medical advice. From December to June, in the following year, when she came to reside in Bath, where applied in succession, poultices, strappings, nitrate of silver in solution and substance, Bates' copper lotion, a double ligature, potassa fusa, and finally the caustic paste, while her failing powers were attempted to be sustained by a generous diet, with quinine and steel. At the time she came to Bath, the tumour had attained the size of a pullet's egg, but was circular in form and much elevated; its surface was smooth, of a purple chocolate colour, and bled upon the slightest touch, while the edges of the surrounding skin were hard and everted; and in the integuments, for four or five inches round the tumour, were scattered several smaller ones, firm and rather insensible, resembling in colour and shape the original at the time it was first noticed. The glands in the upper part of the thigh were enlarged and tender; the patient's complexion, naturally dark, had acquired the sallow leaden hue, which usually accompanies malignant disease; her flesh and strength were gradually decreasing, her appetite was bad, and her nights rendered sleepless by pain.

The obviously malignant character of the tumour, and the state of the femoral glands, forbidding the doubtful resource of amputation, the woman was directed to apply moistened linen to the part, and to take three times a day twenty drops of Brandish's alkaline solution, combined with cascarilla, with the view, if possible, of causing some renewal of appetite and strength. For the first five weeks that this treatment was pursued, no perceptible change took place, either in the tumour or health; but in the sixth week there was an evident improvement in both, and when the patient left Bath, in September, she had recovered her appetite and strength, and her complexion had nearly regained its natural colour, while the tumour, contracted to the level of the neighboring skin, had firmly cicatrized, many of the surrounding tubercles had disappeared, and the glands in the thigh were much reduced in size.

The woman remained well, both as to her foot and general health, up to the end of May in this year, when, though no change appeared in the former, her appetite and strength began to flag. On the 15th of June she was seized with vomiting and purging, followed by pain in the region of the liver, and jaundice, and she died on the 11th of July. During the month which preceded her dissolution, the femoral glands had attained the size of a large hen's egg; and many small, firm, pale tumours, appeared beneath the skin covering the thorax and abdomen, some of which were accompanied by enlarged veins, and small extravasation of blood.

An examination was made eighteen hours after death. There were two small pearly deposits beneath the pleura of the lower part of the left lung; both lungs were studded with cephaloid tubera in every stage of degeneration; two very minute tubercles were deposited in the subserous membrane of the left ventricle of the heart; the liver was much enlarged, and throughout was crowded with innumerable cephalomatous tubera, varying in size from that of a small shot to that of a pullet's egg in every stage of growth, and all more or less deeply tinged with yellow ; the substance of the liver was gorged with bile, and in the vicinity of the larger tumours shewed much vascular congestion. In the sub-mucous tissue of the ileum were two purple, oval, flattened tumours; the inguinal glands were connected with a still larger mass, lying behind Poupart's ligament ; both were of a firm texture, and of a uniform white colour.

The paper was illustrated by coloured drawings of some of the morbid growths in the lungs, liver, and intestines, made by Mr. Hensley, as well as a beautifully-coloured cast of one of the tumours in theileum.—*Prov. Med. Surg. Journ.*

